# Introduction of national guidelines for restrictive blood transfusion threshold for hip fracture patients––a consecutive cohort study based on complete follow-up in national databases

**DOI:** 10.1186/s13018-018-0828-8

**Published:** 2018-05-18

**Authors:** Bjarke Viberg, Per Hviid Gundtoft, Jesper Schønnemann, Lasse Pedersen, Lis Røhl Andersen, Kjell Titlestad, Carsten Fladmose Madsen, Jens Lauritsen, Søren Overgaard

**Affiliations:** 1 Department of Orthopaedic Surgery and Traumatology, Kolding Hospital – part of Hospital Lillebaelt, Sygehusvej 24, 6000 Kolding, Denmark; 2 Department of Orthopaedic Surgery and Traumatology, Odense University Hospital, J. B. Winsløws Vej 4, 5000 Odense C, Denmark; 3 Department of Clinical Research, University of Southern Denmark, Campusvej 55, 5230 Odense M, Denmark; 4 Institute of Regional Health Research, University of Southern Denmark, Campusvej 55, 5230 Odense M, Denmark; 5 Department of Orthopaedic Surgery and Traumatology, Hospital of Southern Jutland, Kresten Philipsens Vej 15, 6200 Aabenraa, Denmark; 6 Department of Orthopaedic Surgery and Traumatology, Hospital of South West Jutland, Finsensgade 35, 6700 Esbjerg, Denmark; 7 Department of Clinical Immunology, Odense University Hospital, J. B. Winsløws Vej 4, 5000 Odense C, Denmark

**Keywords:** Hip fracture, Transfusion, Restrictive, Liberal, Mortality

## Abstract

**Background:**

Randomized controlled trials have demonstrated that a restrictive red blood cell (RBC) transfusion strategy lowers transfusion frequency without affecting mortality. However, the external validity of these trials has not been tested in a large cohort. The purpose was to estimate the effect of introducing a National Clinical Guideline (NCG) for a restrictive hemoglobin transfusion threshold on transfusion frequency and mortality in hip fracture patients > 65 years old.

**Methods:**

A consecutive cohort study of hip fracture patients > 65 years old residing in the southern region of Denmark was conducted using prospectively gathered data from registers during two separate 1-year time periods. The first period from October 1, 2012, to September 30, 2013, included 1494 patients and used a liberal transfusion threshold, whereas the second period from October 1, 2015, to September 30, 2016, including 1414 participants used a restrictive threshold from the NCG. Participant data for age, sex, body mass index, Charlson Comorbidity Index, time to surgery, and death were retrieved from the Danish Interdisciplinary Registry of Hip Fractures and were merged with RBC transfusion and medication data extracted from the Danish Transfusion and Odense Pharmacoepidemiological Databases, respectively. Cox proportional hazards models were used to test relative mortality risk for the restrictive group compared with the liberal group at 30 and 90 days.

**Results:**

Overall RBC transfusions decreased from 42 to 30% (*p* < 0.001). The 30-day mortality rate (95% CI) was 9% (8;11) in the restrictive group and 13% (11;14) in the liberal group (*p* < 0.008), whereas the adjusted relative mortality risk was 0.72 (0.57;0.91). The 90-day mortality rate was 15% (13;17) in the restrictive group and 19% (17;21) in the liberal group, whereas the adjusted relative mortality risk was 0.78 (0.65;0.94).

**Conclusion:**

These data suggest that the introduction of an NCG on restrictive blood transfusion leads to lower transfusion frequency in hip fracture patients > 65 years old. Even though this reduction is associated with decreased mortality at both 30 and 90 days, it may be explained by other issues than restrictive transfusion strategy. There has been an improvement in the mortality of hip fracture patients in Denmark, and we suggest that a restrictive transfusion strategy does not lead to increased mortality.

## Background

Blood loss leading to anemia commonly occurs in hip fracture patients, resulting in approximately half of them receiving RBC transfusions [1, 2]. RBC transfusions are associated with complications such as transfusion-associated circulatory overload, acute hemolysis, and acute lung injury. Importantly, these and other events increase patient morbidity and mortality [3].

Accordingly, in attempts to avoid transfusion-associated complications, a restrictive RBC transfusion strategy has been introduced using a hemoglobin threshold of 8 g/dL as opposed to the liberal 10 g/dL. It is claimed that this approach does not affect mortality, functional recovery, or postoperative morbidity in hip fracture patients [4]. Nevertheless, the previous Cochrane review [4] on this topic demonstrated low-quality evidence specific to hip fracture patients, whereas a more recent Cochrane review [5] referencing a broader range of clinical specialties found that compared with the liberal transfusion threshold, a restrictive hemoglobin threshold of 7 to 8 g/dL decreased the RBC proportion by 43% while also demonstrating evidence supporting no significant impact on 30-day mortality or morbidity. A recent systematic review [6] also suggests that patients with cardiovascular disease undergoing non-cardiac surgery should be set at a threshold of 8 g/dL since a lower threshold may not be safe.

While the National Institute for Health and Care Excellence (NICE) guidelines from 2015 [7] recommend a restrictive transfusion threshold, it is important to note that this is based on very low- to low-quality evidence. Because the NICE guidelines are based exclusively on randomized controlled trials (RCT), there is an additional need for data based on pragmatic trials such as those involving population studies without exclusion of individuals. Therefore, this study aimed to estimate the effect of introducing an NCG for a restrictive hemoglobin transfusion threshold on transfusion frequency and mortality in individuals > 65 years of age with a hip fracture.

## Methods

### Study design

This consecutive cohort study included patients demonstrating a hip fracture within two separate 1-year time periods, prior to and following the introduction of the NCG. The first time period was between October 1, 2012, and September 30, 2013, which was the control period (hereafter referred to as the liberal group). The second time period was between October 1, 2015, and September 30, 2016, which was the intervention period adhering to the Danish NCG [8] for blood component transfusion indications (hereafter referred to as the restrictive group). Reporting of data is performed according to the RECORD extension to the STROBE guidelines [9].

### Setting

The present study was performed in four independent public hospitals in the Southern Region of Denmark, encompassing the entire territory consisting of 1.22 million inhabitants. The Danish National Health Service provides tax-supported free healthcare and general hospital care for all Danish citizens [10]. Since it is not typical for acute hip fracture patients to be treated in the few remaining private hospital clinics, but when this does occur, reporting is mandatory, patients included in this study comprise a consecutive and complete series. Prior to the introduction of the NCG in 2014, the blood transfusion limit was 7.2 g/dL for healthy individuals, with a level of 9.7 g/dL used for individual assessment (e.g., age, rate of anemia, clinical condition, and type of anemia) [11]. The NCG from 2014 references 7.0 g/dL as the lowest transfusion limit for any patient or those demonstrating symptoms of anemia and 8.0 g/dL if the patient has chronic heart disease [8]. This is illustrated in Table 1. In addition, the NCG from 2014 also set a limit of 9.0 g/dL for patients demonstrating an acute coronary syndrome.

**Table 1:** Transfusion thresholds for the liberal and restrictive guidelines

	Liberal	Restrictive
Healthy patients	7.2	7.0
Patients with chronic heart disease	9.7*	8.0

*Individual assessment was applied using age, rate of anemia, clinical condition, and type of anemia instead of chronic heart disease

### Participants

The Danish Interdisciplinary Registry of Hip Fracture database was used to identify the study population. This included all patients admitted with a hip fracture (ICD-10 codes DS720, DS721, and DS722) to one of the four public hospitals in the Southern Region of Denmark. Procedure codes [12] were used to categorize the type of surgery.

### Variables

Participant information for age, sex, body mass index (BMI), Charlson Comorbidity Index (CCI) [13], time to surgery, and death were retrieved from the Danish Interdisciplinary Registry of Hip Fractures. The CCI is calculated by using diagnoses reported to the Danish National Patient Register up to 10 years prior to a hip fracture operation. Patient data regarding hemoglobin was retrieved from the department of Clinical Biochemistry and Pharmacology at the Odense University Hospital, which is able to retrieve data from the whole region. Data regarding transfusion with red blood cells (RBC) was retrieved from the Danish Transfusion Database [14]. Data on patient medication, if reimbursed within 100 days prior to admission to hospital, was retrieved from the Odense Pharmacoepidemiological Database [15].

### Data sources

The Danish Interdisciplinary Registry of Hip Fractures is a population-based clinical quality database established in 2003 and includes data on all hip fracture patients aged 65 or older. Reporting of hip fractures is mandatory for all hospital units, and a number of pre- and peri-operative data are prospectively collected, which includes data on quality of patient care [16]. The Danish Interdisciplinary Registry of Hip Fractures database was linked to the Danish National Patient Registry database in order to extract patient information for BMI, CCI, and reoperations [17].

The Danish National Patient Registry database contains data for all hospital admissions since 1977, which includes dates of admission and discharge, diagnoses (ICD 8 1977-1993, ICD 10 1994 onwards) at discharge and surgical procedure dates and codes according to the Nordic Medico-Statistical Committee (NOMESCO) classification [12]. The Danish Interdisciplinary Registry of Hip Fractures database is also linked to the Danish Civil Registration System database containing information on changes in vital status (e.g., death, new civil registration number, lost, no residence) and migration for the entire Danish population dating back to 1968 [10].

The Danish Transfusion Database is also a population-based clinical quality database maintained since 2006 and linked to the Danish National Patient Registry database. The Danish Transfusion Database contains data from blood bank registries, patient administrative systems, and clinical-biochemical registries. The Odense Pharmacoepidemiological Database is a region-specific prescription database, which covers all inhabitants in the Southern Region of Denmark since 2007. The Odense Pharmacoepidemiological Database contains data for any drug prescription written by hospital staff or general practitioners. However, drug records do not include benzodiazepines, other hypnotics, drugs used to promote weight loss or tobacco abstinence, oral contraceptives, certain antibiotics (e.g., quinolones, tetracyclines), and those that are dispensed over the counter.

### Bias

For this study, data has been included for medication as well as comorbidities in attempts to minimize biases by indication. However, we acknowledge that we are not able to include information on clinical condition nor rate and type of anemia, which are potentially relevant data omissions in terms of RBC transfusion.

### Study size

The survival sample size for finding a reduction of RBC transfusion events yielded 1112 patients for each group. Event for RBC transfusion was set to 50% [1], censoring including death was set to 15%, and minimal clinical difference was set to a hazard ratio of 1.2.

### Quantitative variables

Age was categorized as 65–74, 75–84, and ≥ 85 years old. Three comorbidity levels were defined: a score of 0 (low) was given to patients with no previous record of diseases included in the CCI, a score of 1–2 (medium), and a score of 3 or more (high). Pharmacotherapy use was categorized using dichotomous values (yes/no) for NSAIDs, antihypertensives, glucocorticoids, antidepressants, statins, anticoagulants, and immunosuppressants.

### Statistics

The study population was divided into two comparison groups: group 1 was the restrictive transfusion group following implementation of the NCG (time period October 1, 2015–September 30, 2016), and group 2 was the control group defined as the liberal transfusion group (time period October 1, 2012–September 30, 2013). The statistician was blinded to the groups’ time periods. We described the study population according to the distribution of patients’ characteristics, tabulating the numbers and percentages of patients. Fisher’s exact test was used to evaluate differences between groups for variables of interest. Two-tailed significance was determined using an alpha level set at 0.05.

Mortality at 30 and 90 days was assessed using Kaplan–Meier analyses, with all patients followed until death, emigration, or end of the follow-up period. Relative mortality risk for the intervention group compared with the control group at 30 and 90 days was estimated using Cox proportional hazards modeling. The proportional hazard assumption was tested via log-minus-log plots and was not violated. We adjusted models for age (i.e., 65–74; 75–84, ≥ 85 years), sex, CCI (i.e., 0, 1–2, ≥ 3), and type of surgery (e.g., cannulated screws, sliding hip screw, intramedullary nail, and hip arthroplasty). All outcomes were estimated with a 95% confidence interval (CI).

### Data access, linkage, and cleaning methods

The authors had complete access to aggregated data from the Danish Interdisciplinary Registry of Hip Fractures, Danish Transfusion Database, and Odense Pharmacoepidemiological Database. The civil registration system contains data for individual unique and unchangeable civil registration numbers, which are assigned to all Danish citizens at date of birth or immigration. The civil registration number is recorded in all Danish registers, which allows for unambiguous linkage between registers on an individual basis. Furthermore, the civil registration systems hold information on date of birth, sex, emigration, and death. The prevalence of disappearing persons is 0.3%, which allows for almost complete follow-up for all patients [10].

## Results

The dataset from the Danish Interdisciplinary Registry of Hip Fractures yielded 1494 patients stratified to the liberal group and 1414 for the restrictive group (Table 2). The two groups did not differ for age (*p* < 0.766), whereas there were more males in the restrictive group (*p* < 0.015). There were no group differences for comorbidities (*p* < 0.80) or type of fracture (*p* < 0.851), whereas more intramedullary nails were used in the restrictive group (*p* < 0.001). The surgical delay was also longer for the restrictive compared with the control group (*p* < 0.001).

**Table 2:** Demographic data of the study population and divided by the two cohorts

	Study population		Liberal group		Restrictive group		
	*n*	*%*	*n*	*%*	*n*	*%*	*p<*
Total	2908	100	1494	100	1414	100	
Age							
65–74	590	20	308	21	282	20	
75–84	1018	35	514	34	504	36	0.766
≥ 85	1300	45	672	45	628	44	
Sex							
Female	2.014	69	1065	71	949	67	0.015
Male	894	31	429	29	465	33	
CCI							
None (0)	1019	35	526	35	493	35	0.800
Low (1–2)	1238	43	641	43	597	42	
High (≥ 3)	651	22	327	22	324	23	
BMI							
< 18.5	278	10	153	10	125	9	
18.5–24.9	1269	44	645	43	624	44	
25–29.9	686	24	341	23	345	24	0.433
≥ 30	210	7	102	7	108	8	
Missing	465	16	253	17	212	15	
Prescription medicine							
NSAID	223	8	137	9	86	6	0.002
Diabetes	330	11	166	11	164	11	0.66
Anticoagulant	1191	41	620	42	571	41	0.58
Antihypertensive	1457	50	757	51	700	49	0.58
Statin	720	25	356	24	364	26	0.22
Glucocorticoid	172	6	89	6	83	6	0.94
Depression medicine	888	31	486	33	402	29	0.02
COPD medicine	399	14	209	14	190	13	0.69
Type of fracture							
Femoral neck	1693	59	875	59	818	58	0.851
Pertrochanteric	948	32	480	33	468	33	
Subtrochanteric	267	9	139	9	128	9	
Type of surgery							
Cannulated screws	383	13	206	14	177	13	0.001
Sliding hip screw	677	23	404	27	273	19	
Intramedullary nail	787	27	357	24	431	30	
Arthroplasty	1018	35	506	34	512	36	
Miscellaneous	42	1	21	1	21	1	
Time to surgery							
< 24 h	2050	72	1104	74	946	69	
24–48 h	636	22	317	21	319	23	0.001
> 48 h	180	6	65	4	115	8	

*NSAID* nonsteroidal anti-inflammatory drug, *COPD* chronic obstructive pulmonal disease

In the period following the introduction of the NCG, overall RBC transfusion proportion was reduced from 42 to 30% (*p* < 0.001) (Table 3). One hospital decreased transfusion rates from 43 to 19%, whereas another hospital did not lower it at all. The mean hemoglobin level at admission was 12.3 (12.2;12.3), with no group differences (*p* < 0.540) (Table 4). At the time of first transfusion, the liberal group demonstrated a mean (95% CI) hemoglobin level equal to 9.1 (9.0;9.2), which was compared with the restrictive group at 8.5 (8.2;8.4) (*p* < 0.001). The transfusion threshold differed between the two periods, as 19% of the RBC transfusion was given at a hemoglobin level < 8.0 g/dL in the liberal period compared with 47% in the restrictive period (Table 5).

**Table 3:** Patients admitted and receiving red blood cell transfusion divided by hospital and group

	Liberal group			Restrictive group			
	Admitted	Transfused *n*	Transfused % (95% CI)	Admitted	Transfused *n*	Transfused % (95% CI)	*p* < for transfusion %
Total	1494	628	42 (40;45)	1414	430	30 (28;33)	0.001
Hospital 1	640	268	42 (38;46)	577	145	25 (21;29)	0.001
Hospital 2	312	135	43 (38;49)	283	99	35 (29;41)	0.039
Hospital 3	263	113	43 (37;49)	233	44	19 (14;24)	0.001
Hospital 4	279	112	40 (34;46)	321	142	44 (39;50)	0.311

**Table 4:** Mean hemoglobin level (g/dl) at admission and prior to first red blood cell transfusion by group

	All patients	Liberal group	Restrictive group	
	Mean (95% CI)	Mean (95% CI)	Mean (95% CI)	*p<*
Total, admission	12.3 (12.2;12.3)	12.3 (12.2;12.4)	12.3 (12.2;12.4)	0.54
Total, first transfusion	8.8 (8.7;8.9)	9.1 (9.0;9.2)	8.5 (8.2;8.4)	0.001

**Table 5:** Hemoglobin level at time of first red blood cell transfusion by group

Hemoglobin level	Overall		30-day mortality	Liberal group		Restrictive group		
g/dL	*n*	%	%	*n*	% (95% CI)	*n*	% (95% CI)	*p*<
< 7.0	64	6	14	19	3 (2;4)	45	10 (8;13)	0.001
7.0–7.9	260	25	13	103	16 (13;19)	157	37 (32;41)	
8.0–8.9	339	32	11	207	33 (29;37)	132	31 (26;35)	
> 9.0	345	33	21	254	40 (37;44)	91	21 (17;25)	
Missing	50	5		45	7	5	1	
Total	1058	100		628	100	428	100	

In a subgroup analysis for patients demonstrating a hemoglobin level between 7.0 and 8.9 g/dL (*n* = 1101), which constituted patients with a possible indication for RBC transfusion, 80.7% received an RBC transfusion in the liberal group compared with 58.6% for the restrictive group.

Overall, 30-day mortality was 11% (10;12); in the restrictive group, it was 9% (8;11), and in the liberal group, it was 13% (11;14) (*p* < 0.008). The crude relative mortality risk at 30 days was 0.80 (0.56;1.12) for the restrictive group compared with the liberal group. However, after adjusting for age, sex, CCI, medication, time to surgery, and type of surgery, the relative mortality risk was 0.72 (0.57;0.91). Overall, 90-day mortality was 17% (16;10); in the restrictive group, it was 15% (13;17), and in the liberal group, it was 19% (17;21). Comparing the restrictive to the liberal group, the crude relative mortality risk was 0.91 (0.69;1.19), whereas the adjusted relative mortality risk was 0.78 (0.65;0.94).

In the subgroup analysis for patients demonstrating a hemoglobin level between 7.0 and 8.9 g/dL (*n* = 1101), there was a 30-day mortality proportion equal to 13% (0.10;0.16) in the restrictive period compared with 10% (0.08;0.13) for the liberal period.

## Discussion

Using a population-based study design of hip fracture patients, the present study reported on transfusion frequency and mortality following the introduction of the NCG on restrictive blood transfusion. These data suggest that the lower hemoglobin threshold for RBC transfusion does not increase mortality for older patients with a hip fracture. This observation is consistent with the current literature suggesting there is no difference in patient outcomes between restrictive and liberal treatment strategies [4]. One RCT [18], however, has demonstrated a higher mortality with the restrictive treatment strategy, but this was only for nursing home patients and only per protocol analysis, not the intention-to-treat analysis. This particular RCT is therefore restricted to a small subgroup of hip fracture patients. In contrast, we found a decreased mortality in the restrictive period, which was for all patients, including those with a hemoglobin > 9.0 g/dL. In our data, we see a baseline difference in time to surgery, which is longer in the restrictive group. This may be due to the introduction of new oral anticoagulants (NOAC), which is usually equivalent to a 2-day postponement of surgery to reduce operative bleeding [19]. An increased time to surgery is associated with an increased mortality risk [20], which therefore could lead to an increased risk of mortality in the restrictive group. However, an important bias for mortality is the continuous work for improvement in mortality, and the lowered mortality may therefore lie in other unmeasured aspects since the mortality for hip fracture patients has improved over the last 35 years [21]. An important factor in improving the mortality for hip fracture patients is a multidisciplinary approach [22], and especially an integrated orthogeriatric care [23]. We therefore not interpret the demonstrated decreased mortality as a direct cause to the introduction of a restrictive transfusion strategy.

Being a cohort study, there may be uncontrolled bias. There are several baseline differences which could constitute significant biases. One is the slightly higher proportion of females in the restrictive group, with two retrospective cohort studies [24, 25] finding an odds ratio of 1.5 for females receiving RBC transfusion compared to males. In contrast, Johnston et al. [26] demonstrated in a cohort study of 3625 patients that males had a hazard ratio of 1.8 upon receiving an RBC transfusion compared with females, whereas three other studies did not observe any difference in risk associated with receiving RBC transfusions between males and females [27–29]. Therefore, we suggest that differences between the male and female proportion in this study does not constitute meaningful bias. A second baseline difference is a higher proportion of intramedullary nails in the restrictive group. A shift from sliding hip to intramedullary nails are also seen in other Scandinavian countries [30, 31] resulting in a lower reoperation for complex pertrochanteric fractures [32]. A higher use of intramedullary nails in the restrictive group may increase the likelihood of having a transfusion [24, 25, 28, 33]. This would potentially lead to a slightly higher transfusion proportion in the restrictive group. Accordingly, while the difference in transfusion proportion between the groups could be due to different baselines, we are certain that the reason for a lower RBC transfusion proportion in the restrictive group is due to the lower hemoglobin threshold.

Our large cohort study confirms that a restrictive RBC transfusion threshold does not affect mortality and even though the included studies are of low-quality, a meta-analysis demonstrates no difference in functional recovery or postoperative morbidity in hip fracture patients when comparing restrictive and liberal transfusion thresholds [4]. So why hold on to a liberal transfusion threshold? RBC transfusions are not without complications and specific complications such as transfusion-associated circulatory overload (TACO), acute hemolysis, and transfusion-related acute lung injury (TRALI) can all cause further morbidity and mortality [3]. There may even be a higher association with infections when treated with RBC transfusion [34]. These considerations in combination with our findings should lead to a restrictive transfusion threshold for hip fracture patients.

This study has prospectively collected data from several databases and has precise longitudinal follow-up data due to unique civil registration numbers. This is a major strength since the study is performed on all hip fracture patients and therefore reflects the true clinical value of a restrictive RBC transfusion strategy.

## Conclusion

These data suggest that the introduction of an NCG on restrictive blood transfusion leads to lower transfusion frequency in hip fracture patients > 65 years old. Even though this reduction is associated with decreased mortality at both 30 and 90 days, it may be explained by other issues than restrictive transfusion strategy. There has been an improvement in the mortality of hip fracture patients in Denmark, and we suggest that a restrictive transfusion strategy does not lead to increased mortality.

## Abbreviations

BMI: Body mass index
CCI: Charlson Comorbidity Index
CI: Confidence interval
NCG: National Clinical Guideline
RBC: Red blood cell
RCT: Randomized controlled trial
